# Dimensional Structure of Parent–Child Emotion Dialogues in Families Exposed to Interpersonal Violence: Associations with Internalizing, Externalizing and Trauma Symptoms

**DOI:** 10.1177/08862605251322812

**Published:** 2025-03-15

**Authors:** J. C. de Schipper, M. M. Overbeek, M. H. M. de Moor

**Affiliations:** 1 Vrije Universiteit Amsterdam, The Netherlands; 2 Amsterdam Public Health Research Institute, The Netherlands; 3 ARQ National Psychotrauma Centre, Diemen, The Netherlands; 4 Erasmus University Rotterdam, The Netherlands

**Keywords:** parent–child emotion dialogue, dimensions in emotion dialogue, child behavior problems, trauma outcomes, school age

## Abstract

The way in which parents discuss children’s past emotional events with them is associated with various outcomes in children, such as emotion regulation skills and behavior problems. For children growing up with adverse experiences such as witnessing domestic violence, it is particularly important to understand the link between emotion dialogues and child functioning, because parents’ guidance in dialogues about emotional events may be hampered or suboptimal. Previous studies on parent–child emotion dialogues using the Autobiographical Emotional Events Dialogues (AEED) measure usually focused on composite scores, failing to take into account the various aspects of parent–child emotion dialogues. Therefore, we examined (a) whether a multidimensional structure in the 16 quality rating scales of the AEED can be found in two samples of trauma-exposed and nonexposed parent–child dyads (*N* = 234, child age 4–13 years), (b) whether the identified dimensions predict child internalizing, externalizing and trauma symptoms. Principal components analyses showed multidimensionality in emotion dialogues with two factors that replicated across samples, labeled sensitive guidance & child cooperation and closure/resolution. Regression analyses showed that closure/resolution predicted fewer internalizing symptoms. When dyads ended dialogues about children feeling sad, scared, or angry in a more constructive way, children showed fewer internalizing symptoms. Sensitive guidance & child cooperation predicted less externalizing and trauma symptoms in children. Our study suggests that the degree of positive closure of a dialogue about negative emotions might be a specific aspect of parent–child emotion dialogue related to child outcomes that might have potential clinical relevance.

The way in which parents discuss children’s past emotions with them is associated with various outcomes such as children’s understanding of different emotions (Laible, 2011; Laible & Song, 2006; Valentino et al., 2015), their emotion regulation (Curtis et al., 2020; Ellis et al., 2014; Speidel et al., 2019), and child behavioral problems (Hernandez et al., 2018; McDonnell et al., 2022; Sales & Fivush, 2005). Previous studies have further shown that the quality of parent–child discussion about children’s past emotional events may act as a mediator in the association between children’s degree of exposure to adversities such as child maltreatment, interparental violence and child sexual abuse, and child socioemotional outcomes (Speidel et al., 2019; Valentino et al., 2015). In situations of interpersonal violence, parents’ guidance in dialogue about emotional events may be hampered or suboptimal due to the role they themselves might play in these events or due to their own emotion regulation difficulties (Dyb et al., 2003; Lagdon et al., 2014; van Duin et al., 2018).

Salmon and Reese (2015) state that reminiscing conversations in parent–child dyads about children’s emotional experiences are likely to support children in remembering and understanding their emotions, and as a consequence, children might be better able to manage difficult emotions. Reminiscing dialogue with children exposed to interpersonal violence might therefore be pivotal to understand their complex emotional experiences. In line with this argument, trauma-focused clinical practice for children contains building blocks in which the therapist is helping children to express and understand their feelings and manage complex emotions (Cohen et al., 2017). Salmon and Reese (2015) argued that more effort is needed to understand how parent–child emotion dialogues may help or hinder child functioning, particularly in families exposed to interpersonal violence.

From an attachment perspective, parents serve as a secure base for their children (Ainsworth et al., 1971; Bowlby, 1988). At a young age, parents are usually a secure base in a more physical way (Ainsworth, et al., 1971; Bowlby, 1988) while in older children, they provide a secure base in a more psychological way, through talking with their children about their experiences, being available and supporting them (Kerns, 2008; Marvin & Britner, 2008), thus providing a psychological secure base from which to explore (Bowlby, 1988). Through the lens of this attachment perspective, Koren-Karie et al. (2003a) developed the Autobiographical Emotional Events Dialogue (AEED) to assess patterns in parent–child emotion dialogues about children’s emotional events. In the AEED task, the parent is asked to discuss with the child four situations in which the child felt happy, sad, scared, and angry. By discussing emotional events, parents may help their children understand and process their feelings regarding the world around them. While developing their measure, Koren-Karie et al. (2003b) analyzed that in parent–child emotion dialogues, the concept of the psychological secure base is expressed in the language of both parent and child. In different age groups, from preschoolers up to adolescents, they identified four categories of parent–child emotion dialogues, that is, one emotionally matched category and three non-matched categories. These categories have been found to be associated with children’s earlier attachment classification in infancy (Hsiao, Koren-Karie, Bailey, & Moran, 2015; Oppenheim et al., 2007).

In emotionally matched dyads, transcripts show that parent and child work together and provide a clear story on the four emotions. Stories might be short, but both parent and child contribute and there is acceptance of each other’s input. Points of strengths are discussed and stories about negative emotions are likely to end with a positive closure (Koren-Karie et al., 2003a, 2003b). In emotionally unmatched dyads, there might, for example, be a lack of coherence in the stories; the emotions discussed might be quite extreme with no completion of the narrative (unmatched exaggerated); or very little is said resulting in no development of the story and of what it meant for the child; (unmatched flat); or there is large inconsistency in the quality and coherence across the four stories or in the parent and the child’s contribution to the story (unmatched, inconsistent).

There are constraints in the use of the AEED categories in statistical analyses. The distribution of the four categories is often unequal, also in clinical samples, which asks for considerable sample sizes. Therefore, the four categories are usually reduced to two categories—emotionally matched versus emotionally unmatched parent–child emotion dialogues. This, however, diminishes the richness of the dialogue patterns observed in dyads and their possible specific roles in child outcomes. As an alternative to the categorical assessment, the 16 rating scales that are also part of the coding manual—(to support the classification)—have been summarized in previous research in composite scores focusing on *sensitive guidance* using the parent scales (Hsiao et al., 2015; Koren-Karie & Aviezer, 2017; Koren-Karie et al., 2008; McDonnell et al., 2022; Overbeek et al., 2022; Speidel, et al., 2019; Valentino et al., 2019, 2022) and *child cooperation and involvement* using the child scales (Hsiao et al., 2015; Koren-Karie & Aviezer, 2017; Koren-Karie et al., 2008; Overbeek et al., 2022). Sometimes a third composite was used, reflecting aspects of the quality of the stories told in the dialogue (Koren-Karie & Aviezer, 2017; Koren-Karie et al., 2008; Overbeek et al., 2022). Composite scores make it easier to analyze associations between aspects of emotion dialogues and child functioning but have similar limitations with regard to constraining the richness in dialogue patterns observed. In addition, in studies that used composite scores, the child rating scales were sometimes left out (i.e., McDonnell et al., 2022; Speidel et al., 2019), whereas the AEED measure is conceptualized as a dyadic measure. Furthermore, there appears to be a conceptual constraint in summarizing the rating scales. The developers argue that kindness and warmth, which relate to sensitivity, are not the core characteristics of an emotionally matched parent–child conversation (Koren-Karie et al. 2003b). Rather, they present the following characteristics as the core aspects of emotionally matched parent–child dialogues: coherence between the story and the emotion discussed; mutual engagement; dialogues usually evolve toward points of strengths and/or a positive closure of negative emotions; as well as maternal structuring and organization of the task on the one hand and child cooperation and elaboration on the other (Koren-Karie et al., 2003a, 2003b; Oppenheim et al., 2007). This suggests that the composite scores that are used in studies are not in complete alliance with the conceptualization of the most important characteristics in emotion dialogues in parent–child dyads according to the AEED measure.

Conceptual analyses of the AEED assessment by the developers (Koren-Karie et al., 2003a, 2003b; Oppenheim et al., 2007) indicate that various aspects of emotion dialogue are relevant for distinguishing between optimal and suboptimal emotion dialogues. This suggests that the AEED rating scales may have a multidimensional structure. There are seven rating scales regarding the parent’s contribution to the dialogue: (a) acceptance: tolerance regarding the child’s contribution; (b) involvement and reciprocity; (c) structuring the dialogue; (d) closure: containment of negative feelings the child discusses; (e) focus on completing the task; (f) boundary dissolution: failure to accept the child’s own perspectives and experiences; and (g) hostility. Parallel with the parent scales, seven rating scales evaluate the child’s contribution to the dialogue: (a) acceptance of parent’s guidance; (b) cooperation; (c) elaboration in the stories; (d) resolution of more difficult topics; (e) focus on the task; (f) boundary dissolution: not maintaining child role, and (g) hostility. The two final scales refer to the quality of the dialogue: adequacy of the stories told about each emotion, and overall coherence in the stories told. In this study, we will explore whether different dimensions of parent–child emotion dialogue can be identified, by examining the multidimensionality of the 16 rating scales of the AEED.

To assess the predictive validity of the dimensional structure in parent–child emotion dialogues, we further aim to study associations between the found dimensions in emotion dialogues and child outcomes. Previous studies showed that parent contributions to emotion dialogues were linked with children’s externalizing symptoms (McDonnell et al., 2022; Sales & Fivush, 2005) and internalizing symptoms (Brumariu & Kerns, 2015; Hernandez et al., 2018; McDonnell et al., 2022; Sales & Fivush, 2005). Overbeek et al. (2022) found that parent–child emotion dialogues of lower quality were associated with more post-traumatic stress symptoms in children. In addition, maladaptive parenting behavior has been linked with post-traumatic stress symptoms (Valentino et al., 2010). Therefore, we will explore associations between identified dimensions in parent–child emotion dialogue and child internalizing, externalizing, and trauma symptoms.

To summarize, two research questions will be addressed in this study. First, we will study whether a multidimensional structure in the 16 quality rating scales of parent–child dialogues about emotional events from the AEED measure can be found in two samples of trauma-exposed and nonexposed parent–child dyads. In case we find different dimensions of parent–child dialogue, our second research question is whether these identified dimensions are associated with child internalizing, externalizing, and trauma symptoms.

To address these questions, we used two samples that included children exposed to interpersonal violence (interparental violence and child sexual abuse) and nonexposed children and analyzed the factor structure in these studies separately. For the second question regarding associations of dimensions with child outcomes, data from the two studies were combined.

## Methods

### Samples

#### Sample 1

The first sample of 116 parent–child dyads comes from a study with families exposed to interparental violence (IPV) (Overbeek et al., 2013). In this study, children and their non-violent parents were invited to participate in a group intervention for child witnesses of interparental violence. They were randomly assigned to either a community-based IPV-focused group intervention or a control group intervention with only non-specific intervention factors (such as positive attention and doing fun things). Both interventions consisted of nine sessions, with a maximum of eight children and eight parents participating. The program was standardized and therapists followed a manual for each session. General specific treatment factors in the IPV-focused program for children were, for example, practicing emotion differentiation and regulation, and coping skills (Overbeek et al., 2012; Overbeek et al., 2013). Trauma-specific treatment factors were, for example, coping skills focused on IPV and inviting to share experiences regarding IPV. In the IPV-focused program for parents, general specific treatment factors were psycho-education in general and parenting skills. Trauma-specific factors were among others: psycho-education regarding IPV, taking the child’s perspective with regard to IPV, and inviting them to share IPV experiences. No specific training on how to talk about emotions with children was included in the program.

The sample included 63 boys (54%) and 53 girls (46%), aged 6 to 13 years old (*M* = 9.41; *SD* = 1.55). A considerable group of children had a non-Dutch immigration background (67%); most of them had a Turkish, Moroccan, Suriname, or Antilles background. For six children, the father was the primary caregiver. In most cases, both partners had used violence in the marital relationship, although women more often reported psychological violence compared to the more physical violence of their (ex-)partners. Of the participating parents, 87% had finished high school education at most and/or middle vocational education. A considerable number of families (64%) had a yearly income below the poverty level (lower than 15.000 euros a year).

#### Sample 2

In the second study, 118 mother-child dyads participated. Half of the children were exposed to interpersonal trauma, of whom 29 to interparental violence (Visser et al., 2016) and 30 to child sexual abuse (van Delft et al., 2018). These children were referred to a child trauma center and matched with a community comparison group (without exposure to interpersonal trauma). The sample included 56 boys (48%) and 62 girls (52%), aged 4 to 13 years old (*M* = 8.31; *SD* = 2.44). Of the participating mothers, 84% had finished high school education at most and/or middle vocational education. A small group of mothers had a non-Dutch immigration background (11%) and 21% of the families had a yearly income below the poverty level. For the analyses regarding child outcomes (based on CBCL data), 90 children were included, due to missing variables (parents who did not fill out the questionnaire).

### Procedure

#### Sample 1

In this study, the AEED was administered at two time points: at posttest after the intervention and at follow-up, 6 months after finishing the intervention. To reduce the potential influence of the intervention on the dimensional structure of the AEED, we analyzed AEED data collected at follow-up. It was possible for parent–child dyads to carry out this task in their native language. For 13 dyads, an interpreter was used for transcribing (parts of) the dialogue. Usually, no interpreter was needed for explaining the task to the parent and child. Overbeek et al., (2013) describe further details of the RCT design and procedures. Ethical approval was received by the Medical Ethics Committee of the VU University medical center in Amsterdam, the Netherlands, METc VUmc 2009/99/NL26649.029.09.

#### Sample 2

This study consisted of four subsamples of children. Two trauma-exposed subsamples were recruited from three outpatient children’s trauma centers across The Netherlands. Parent–child dyads were referred to these centers for assessment and treatment. They were referred by Youth Care Agencies (Visser et al., 2016; van Delft et al., 2018), general practitioners, or mental health care professionals after exposure to child sexual abuse or exposure to interparental violence. For each subsample, a comparison subsample was recruited with no exposure to interpersonal violence. Details of the pretest data of subsamples are reported separately for children exposed to interparental violence (Visser et al., 2016) and for children exposed to sexual abuse (van Delft et al., 2018). Ethical approval was received by the Medical Ethics Committee of the VU University Medical Center in Amsterdam (METc VUmc 2011/101/NL39277.029.12 and METc VUmc 2011/407/NL38753.029.11). We combined the pretests of the four subsamples in our analyses, given their small size.

### Measures

#### Autobiographical Emotional Events Dialogue

A common task to assess the quality of parent–child emotion dialogue is the Autobiographical Emotional Events Dialogue (AEED, Koren-Karie, et al., 2003a). In this task, parent–child dialogues about past events in which the child felt happy, sad, scared, and angry are assessed. Four cards that picture these emotions are used to help parent and child. For each emotion, parents and children are asked to select an event to talk about. Coding was conducted by analyzing the transcript of the dialogue, identifying indicators for the 16 scales throughout the transcript, and then assigning a score for each scale based on the frequency and intensity of these indicators. Parental and child contributions were described with seven scales: (a) Acceptance: parent/child allows the other to express a wide range of emotional themes without defensiveness or judgment; (b) Involvement and cooperation: parent/child is actively engaged in the task and demonstrates genuine interest in the stories; (c) Structuring and elaboration: the parent assists the child in telling a rich and coherent story and the child is able to tell rich and detailed stories; (d) Closure/resolution: stories with negative themes are resolved with a focus on the child’s coping abilities and strengths; (e) Focus: parent and child are focused on the child’s emotions without digressing into irrelevant details or shifting to the parent’s own feelings; (e) Boundary Dissolution: parent and child maintain appropriate roles, avoiding the parent imposing their emotions or becoming overwhelmed by the child’s stories, and the child does not take on a parental role such as promising to protect the parent, and (f) Hostility: parent or child exhibits thematic hostility, anger, or derogation. In addition, one scale was used to describe the Adequacy of the stories that the child and parent had discussed for each emotion, and another scale was used to assess the overall coherence of the dialogue. Every scale was scored from 1 to 9. Higher scores indicate more enactment of the specific behavior. AEED transcripts for sample 1 were double coded by four coders, and for sample 2 single coded by the 2nd author. All coders were trained by one of the developers of this measure, N. Koren-Karie. To improve coding in a sample of trauma-exposed children, all coders together coded 27 transcripts of the sample of trauma-exposed children in consultation with the developer, in addition to the original reliability set. After this additional training, the average ICC between the four coders was calculated and ranged from .74 to .89 (averaged over all 16 scales). Single ICC of the second author was .79 averaged over all 16 scales.

#### Child Symptoms

##### Internalizing and Externalizing Symptoms

The Dutch translation of the Child Behavior Checklist for Children (CBCL; Achenbach & Rescorla, 2001; Verhulst, et al. 1996) was filled out by the primary caregiver (*n* = 206 children). Sum scores were computed for internalizing symptoms (32 items, Cronbach’s alpha for samples 1 and 2 is .85 and .89 respectively) and for externalizing (35 items, Cronbach’s alpha = .91 and .92, respectively).

##### Post-Traumatic Stress Symptoms

Based on research by Sim et al. (2005) and by Milot et al. (2013), 16 items of the CBCL were used as a proxy to assess children’s post-traumatic stress symptoms. Mean scores across items were computed (Cronbach’s alpha = .69 for sample 1 and .89 for sample 2). In these previous two studies, the scales based on the CBCL showed moderate to strong associations (.32 < *r* < .65) with clinical measures for children’s PTS symptoms.

Externalizing symptoms correlated .61 with internalizing behavior and .84 with trauma symptoms and internalizing symptoms correlated .64 with trauma symptoms.

For the second research question, the samples were combined. The descriptives of child symptoms are therefore reported in the results section (prediction of symptoms).

### Statistical Analyses

All analyses were conducted in SPSS version 25. Descriptive statistics were computed for all variables (e.g., means, standard deviations, and correlations). To examine the dimensional structure of the AEED (research question 1), principal component analyses (PCA) with Promax rotation were run for the 16 AEED scales in each sample. Parent and child scales for hostility and boundary dissolution were reverse scored prior to factor analyses, such that higher scores correspond to less hostility and boundary dissolution, to ease the interpretation of the factor loadings (all positive).

Factor solutions were inspected by examining the eigenvalues and the factor loadings (pattern matrix). The number of factors was chosen based on eigenvalues larger than 1. Further, scales were assigned to the factor with the largest factor loading and only if larger than 0.3. Then, the obtained factor solutions were compared across the two trauma-exposed samples to examine whether they would replicate. Finally, sensitivity analyses were carried out. In sample 1 the PCA was also conducted on AEED data from the posttest rather than at follow-up, to examine the robustness of the obtained factor solution in this sample across timepoints. In sample 2, the PCA was also run in the clinical and community comparison group separately, to explore to what extent the factor solution differed in these two groups. The results of these sensitivity analyses are reported in the Supplemental Information.

To examine to what degree the obtained factors predict child internalizing, externalizing, and trauma symptoms (research question 2), the replicated factors (mean scores of the scales that load on each factor) were entered as multiple predictors in a hierarchical regression analysis for each outcome separately in the combined sample (merging the data from sample 1 and 2, to maximize statistical power to detect predictive associations). In the first block of the hierarchical regression analysis, covariates (parental educational level, child age, child gender) that were found to be significantly associated with the outcome were included as predictors. Next, the factor with the largest proportion of explained variance in the PCA was added, followed by all other factors that replicated across samples.

## Results

### Descriptives of the AEED Scales

The means, standard deviations, and ranges of scores of all 16 AEED scales are reported in Table 1 for both samples. The descriptives of the two samples are quite similar. In both samples, the means are around 4 or 5 for most scales, but with large variation in scores spanning (almost) the full possible range of scores from 1 to 9. Exceptions are parental and child focus, which tend to have relatively high means, and parental and child hostility and boundary dissolution have relatively low means. This indicates that on average focus is high and hostility and boundary dissolution are low. For sample 1, the descriptives for the posttest are given in Supplemental Table 1, which are comparable to the descriptives at follow-up. For sample 2, the descriptives for the clinical subsample and the community comparison subsample are provided in Supplemental Table 2, indicating that scores significantly differ between the two groups for all scales except parental focus, child boundary dissolution, and child hostility. In general, scores indicate less optimal patterns of interaction in the clinical subsample.

**Table 1.** Descriptives of the AEED Scales in Two Samples of Parent–Child Dyads.

	Sample 1 (*N* = 116)			Sample 2 (*N* = 118)		
AEED Scales	Min	Max	*M* (*SD*)	Min	Max	*M* (*SD*)
Parental acceptance	3	8	5.38 (1.10)	2	9	5.72 (1.60)
Parental involvement	1	9	5.10 (1.31)	2	8	5.30 (1.55)
Parental structuring	1	9	4.38 (1.37)	1	8	4.46 (1.65)
Parental closure	1	7	4.50 (1.14)	1	8	4.72 (1.54)
Parental focus	3	9	7.32 (1.81)	3	9	7.74 (1.49)
Parental boundary dissolution	1	9	1.85 (1.41)	1	7	1.96 (1.30)
Parental hostility	1	5.5	1.16 (0.59)	1	6	1.18 (0.64)
Child acceptance	1	9	6.07 (1.35)	2	8	5.90 (1.54)
Child cooperation	1	9	5.52 (1.43)	2	8	5.34 (1.63)
Child elaboration	1	8	4.07 (2.01)	1	8	3.61 (1.98)
Child resolution	1	6.5	4.54 (0.91)	2	8	4.76 (1.05)
Child focus	1	9	8.07 (1.36)	2	9	8.14 (1.61)
Child boundary dissolution	1	9	1.53 (1.16)	1	7	1.80 (1.44)
Child hostility	1	9	1.18 (0.88)	1	5	1.21 (0.61)
Adequacy	1	9	4.33 (1.71)	2	9	4.69 (1.97)
Coherence	1	8	3.86 (1.67)	1	8	3.88 (1.92)

*Note.* Higher scores describe more enactment of the specific behavior.

The correlations among the 16 scales in the two samples are provided in Table 2. Regarding the parental scales, large to very large correlations are found among Acceptance, Structuring, and Involvement in both samples (.54 ≤ *r* ≤ .82), and zero to large correlations among the other parental scales (−.01 ≤ *r* ≤.50). For the child scales, also large to very large correlations are found among the child counterpart of the above-mentioned parental scales: Acceptance, Elaboration, and Cooperation in both samples (.61 ≤ *r* ≤.82), and zero to large correlations among the other child scales (.00 ≤ *r* ≤ .52). Further, Parental acceptance, Structuring, and Involvement also correlate highly with Child acceptance, Elaboration, and Cooperation, and with the dyadic scales Adequacy and Coherence (.43 ≤ *r* ≤.88). Large correlations are further noted between parental closure and child resolution in both samples (*r* = .76 and *r* = .66). In Supplemental Table 3, the correlations among the scales are provided for the posttest of sample 1.

**Table 2.** Pearson Correlations among AEED Scales in two Samples of Parent–Child Dyads.

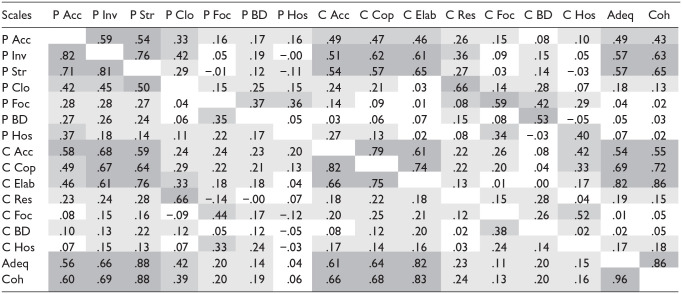

*Note.* Correlations for sample 1 on upper diagonal, and for sample 2 on lower diagonal. P = parental; C = child; Acc = acceptance; Inv = involvement; Str = structuring; Clo = closure; Foc = focus; BD = boundary solution; Hos = hostility; Cop = cooperation; Elab = elaboration; Res = resolution; Adeq = adequacy; Coh = coherence. Please note that *BD and Hos scales* have been *reverse coded*. Dark gray: large to very large effect size (*r* > .50), medium gray: moderate-to-large effect size (.30 < *r* < .50), light gray: small-to-medium effect size (.10 < *r* < .30). All correlations of .18 or lower were not statistically significant (*p* > .05).

### Multidimensional Structure of the AEED

The factor solutions from the PCA in both samples are provided in Table 3. In both samples, the PCA yields four factors with eigenvalues larger than 1, which together explain 72% of the variance in sample 1 and 70% of the variance in sample 2. The first and largest factor found in both samples includes Parental acceptance, Involvement and Structuring, Child acceptance, Cooperation, and Elaboration, and the Dyadic scales Adequacy and coherence. This factor can be labeled sensitive guidance & child cooperation and explains the largest portion of variance (36% in sample 1 and 41% in sample 2). A second factor, that replicates across the two samples, consists of the two parallel scales parental closure and child resolution and is labeled closure/resolution. The two other factors are less consistent across the samples. In sample 1, one factor consists of the child focus and parental and child hostility scales, and the other factor contains parental focus and parental and child boundary solution. In sample 2, however, parental focus, hostility, and boundary dissolution form one factor, while the other factor includes child focus, hostility, and boundary dissolution. Cronbach’s alpha’s for the factor Sensitive Guidance & Child Cooperation were .93 and .92 in samples 1 and 2 respectively, and for closure/resolution .78 and .76.

**Table 3.** Factor Solution of the Principal Component Analysis of the AEED Scales in Two Samples of Parent–Child Dyads.

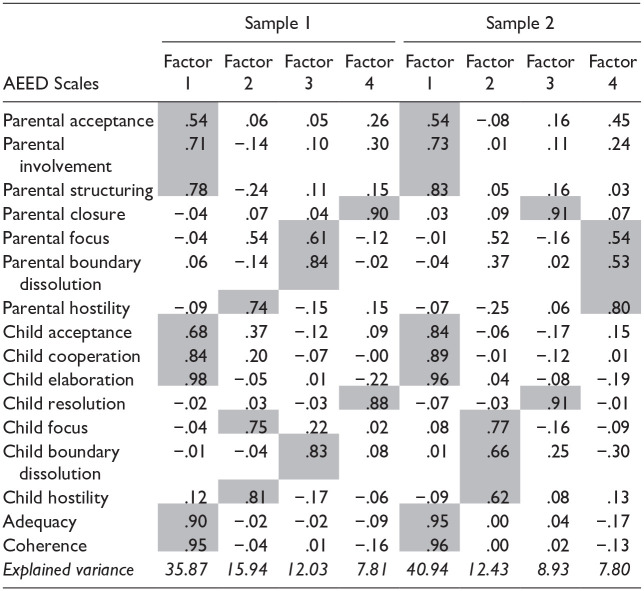

The factors correlate low to moderately high among each other in the two samples. The factor sensitive guidance & child cooperation shows medium correlations with the factor closure/resolution (*r* = .30 in sample 1 and *r* = .38 in sample 2), and small-to-medium correlations with the other factors including the focus, hostility, and boundary dissolution scales (.08 < *r* < .32). Low correlations are found between the factor closure/resolution and the factors including the focus, hostility, and boundary dissolution scales (−.07 < *r* < .19). In Supplemental Table 4, the factor solutions from the PCA at posttest from sample 1 are provided. In Supplemental Table 5, the factor solutions from the PCA from the clinical subsample of sample 2 are provided.

### Prediction of Internalizing, Externalizing, and Trauma Symptoms

#### Replicated Factors as Predictors

The results of the hierarchical regression analyses (*n* = 206), with the replicated factors sensitive guidance & child cooperation (*M* = 4.85, *SD* = 1.37) and closure/resolution (*M* = 4.63, *SD* = 1.07) predicting child internalizing (*M* = 7.59, *SD* = 7.61), externalizing (*M* = 7.81, *SD* = 8.43) and trauma symptoms (*M* = 0.30, *SD* = 0.29), are provided in Table 4. Before entering into the regression analyses, square root transformation was applied for each child outcome to render the data more normally distributed.

**Table 4.** Hierarchical Regression Analyses for the Replicated AEED Factors Predicting Internalizing, Externalizing, and Trauma Symptoms.

Child outcome	B	*SE*	Beta	*t*	*p*	*R* ^2^	*R*^2^ change
Internalizing symptoms							
Step 1						.002	.002
Intercept	2.60	0.37		7.07	<.001		
Sensitive guidance & cooperation	−0.05	0.07	−.05	−0.64	.53		
Step 2						.021	.019*
Intercept	3.23	0.48		6.68	<.001		
Sensitive guidance & cooperation	0.01	0.08	.01	0.13	.90		
Closure/resolution	−0.20	0.10	−.15	−1.98	.050		
Externalizing symptoms							
Step 1						.032*	.032*
Intercept	3.73	0.55		6.78	<.001		
Child age	−0.15	0.06	−.18	−2.58	.011		
Step 2						.054**	.022*
Intercept	4.61	0.68		6.79	<.001		
Child age	−0.16	0.06	−.18	−2.76	.006		
Sensitive guidance & cooperation	−0.17	0.08	−.15	−2.17	.031		
Step 3						.055**	.001
Intercept	4.83	0.78		6.16	<.001		
Child age	−0.16	0.06	−.19	−2.73	.007		
Sensitive guidance & cooperation	−0.15	0.08	−.13	−1.82	.07		
Closure/resolution	−0.06	0.11	−.04	−0.56	.58		
Trauma Symptoms							
Step 1						.019*	.019*
Intercept	0.61	0.07		8.80	<.001		
Sensitive guidance & cooperation	−0.03	0.01	−.14	−2.00	.047		
Step 2						.034*	.015
Intercept	0.71	0.09		7.84	<.001		
Sensitive guidance & cooperation	−0.02	0.02	−.09	−1.23	.22		
Closure/resolution	−0.03	0.02	−.13	−1.75	.08		

* *p* < .05, **p < .01.

Closure/Resolution but not Sensitive Guidance & Child Cooperation was a significant predictor of child internalizing symptoms, explaining 1.9% of the variance in symptoms (*F* [1,203] = 3.90, *p* = .0496) indicating a small effect. In dyads who were better able to end stories about negative emotions in a positive or strength-focused way, children showed less internalizing behavior.

After controlling for child age, Sensitive Guidance & Child Cooperation significantly predicted child externalizing symptoms, explaining 2.2% of the variance (*F* [1,202] = 4.77, *p* = .030). In dyads with more child cooperation and with parents showing more sensitive guidance, children showed less externalizing behavior. However, this association did not remain statistically significant after including the factor closure/resolution. The final standardized regression coefficient of sensitive guidance & child cooperation remained small after the inclusion of the factor closure/resolution and was slightly attenuated from −.15 to −.13.

For trauma symptoms, similar results were obtained as for externalizing symptoms. Sensitive Guidance & Child Cooperation significantly predicted child trauma symptoms, explaining 1.9% of the variance (*F* [1,204] = 3.98, *p* = .047), but this association was no longer statistically significant after including closure/resolution. The standardized regression coefficient was slightly attenuated from −.14 to −.09 for sensitive guidance & child cooperation after the inclusion of closure/resolution.

In addition to the predictions of the replicated factors sensitive guidance & child cooperation and closure/resolution, we explored whether the six parental and child scales focus, boundary dissolution, and hostility, additionally predicted child internalizing, externalizing, and trauma symptoms. The results are presented in Supplemental Table 6. In brief, only a significant positive association was found between parental boundary dissolution and externalizing symptoms (*b*[*SE*] = 0.19 (0.09); *t* = 2.09, *p* = .038). The variance in externalizing symptoms uniquely explained by parental boundary dissolution was 2.0% (*F* [1,195] = 4.36, *p* = .038), indicating a small effect. In dyads with parents who show more signs of boundary dissolution, children show more externalizing behavior, after adjusting for child age, sensitive guidance & child cooperation, closure/resolution, and the other maladaptive AEED scales. None of the other subscales significantly predicted internalizing, externalizing, and trauma symptoms, in addition to the replicated factors sensitive guidance & child cooperation and closure/resolution. The total variance explained when adding the six subscales was 4.0% for internalizing symptoms, 5.6% for trauma symptoms (both nonsignificant), and 10.5% for externalizing symptoms (*p* = .009).

## Discussion

Conceptual analyses of parent–child emotion dialogues in the *Autobiographical Emotional Event Dialogues* assessment (Koren-Karie et al., 2003a, 2003b), suggest that for children, different aspects in these dialogues are relevant to experience a secure base from which to explore their emotions. Therefore, we examined whether the rating scales of the AEED might show a multidimensional structure. In addition, we analyzed the extent to which the identified dimensions predict internalizing, externalizing, and trauma symptoms. In two samples of parent–child dyads with one-third of the children having a migration background and 75% having been exposed to interpersonal violence, PCA identified four dimensions in the AEED. The first and largest dimension identified in both samples was labeled Sensitive Guidance & Child Cooperation. This dimension consisted of parent and child scales assessing acceptance, involvement, and structuring as well as the two scales describing the quality of the dialogue (adequacy and coherence) and describing more adaptive ways of interacting in emotion dialogues. High scores on this dimension indicate high involvement and cooperation of both parent and child, resulting in clear and coherent stories about each of the four emotions discussed. A second dimension of parent–child emotion dialogue found in both samples was closure/resolution. Parent–child dyads with high scores on closure/resolution were better able to end stories about negative emotions in a positive way focusing on strategies to cope with more complex and negative feelings. For the two identified dyadic factors, it was found that higher scores on closure/resolution predicted less internalizing symptoms in children. Furthermore, higher scores on the sensitive guidance & child cooperation factor predicted less externalizing symptoms and less trauma symptoms. The remaining scales of the AEED (child and parent focus, hostility, and boundary dissolution) did not cluster in a consistent way in the two samples. Furthermore, these scales overall did not predict child outcomes, with the exception of parental boundary dissolution predicting more child externalizing symptoms.

The dyadic factor closure/resolution differs from earlier concepts in parent–child emotion dialogue (e.g., McDonnell et al, 2022; Koren-Karie et al., 2003a, 2003b), in that it identifies a specific dyadic pattern related to (supporting) children’s understanding and coping with more complex or negative feelings. A high score on closure indicates that the parent acknowledges the negative feelings of the child and does not ignore or belittle the story of the child. Furthermore, parents focus on a positive ending by either stressing the child’s strength in dealing with the situation, or they might point out that the situation has ended now. High resolution indicates that children end their story about a negative emotion in a positive way, for example, by how they coped well with the emotion. This factor explained a small part of the variance in child internalizing behavior, supporting the predictive validity of this new construct. This newly identified dimension may provide us with more specificity about dyadic interaction patterns in emotion dialogues, in particular for families exposed to interpersonal violence.

Furthermore, in families with higher parental sensitivity in emotion dialogues and higher cooperation in children, fewer externalizing symptoms were reported as well as fewer trauma symptoms. This is partly in line with McDonnell et al. (2022) who found that mothers’ sensitive guidance was associated with less externalizing and internalizing behavior. Thus, partial support was found for the predictive validity of the dyadic sensitive/engaged factor and the dyadic closure/resolution factor in predicting internalizing, externalizing, and trauma symptoms in children.

The six scales of the AEED referring to more maladaptive patterns, that is, hostility, boundary dissolution, and lack of focus, formed two additional factors in the factor solution of the samples, but with different constellations in each sample. The lack of consistency in maladaptive factors in our two samples might result from the limited variance in these scales. Given this limited variance, it is too early to conclude that there is no need to further study maladaptive patterns in parent–child emotion dialogues.

Our study suggests that using composite scores of the AEED scales in studies with trauma-exposed children might not fully cover the richness of the data that is collected with the 16 scales. In clinical practice, helping parents to discuss difficult emotions with their children may help their children validate and cope with their experiences and complex feelings. Intervention studies showed that training parents in supporting children in elaborative reminiscing resulted in higher maternal support in emotion communication and richer parent–child discussions of emotional events (Valentino et al., 2013, 2019). A one-year follow-up of this focused reminiscing training for mothers in maltreating families indicated that improvement in maternal guidance in emotion dialogues mediated the effect of the training on the reduction of child problem behavior (Valentino, et al., 2022). In a study on trauma-focused cognitive behavior therapy for children, support from parents during children’s trauma narratives was associated with a decrease in children’s internalizing symptoms, whereas caregiver avoidance and blame resulted in worsening of children’s symptoms over the course of treatment (Yasinski et al., 2016). These intervention studies suggest that further study and validation of the factors of closure/resolution and sensitive guidance & child cooperation as well as the maladaptive scales of the AEED may increase our understanding of the role of parent–child emotion dialogues in child and family outcomes in clinical samples.

### Strengths and Limitations

To our knowledge, this is the first study exploring the factor structure of the scales of the AEED measure. For replication of the factor structure, two samples were used, including community-based and referred children exposed to interparental violence or sexual abuse as well as nonexposed children. Although in sample 1, a majority of the children had a non-Dutch migration background, and in both samples, children were exposed to different types of violence/abuse, a limitation of our study was that subgroups of participants were too small to examine potential differences related to migration background or type of violence. Because we included both clinical and nonclinical samples, a second limitation was that we assessed child trauma symptoms using a scale from a general child behavior questionnaire that is not thoroughly validated for this purpose. Furthermore, behavioral problems of children were reported by the primary caregiver who also participated in the emotion dialogue task. Finally, generally low frequencies on maladaptive scales may have limited the consistent identification of maladaptive dimensions in parent–child emotion dialogues. Future research may focus on further validation of the closure/resolution factor, in more diverse samples with respect to background, exposure to violence, and other trauma, and with respect to community, clinical, and nonclinical samples. Behavioral problems may be assessed through reports from all primary caregivers as well as teachers.

## Conclusion

Our factor analyses in two samples that included children exposed to interpersonal violence showed that in addition to a broad factor of parental sensitive guidance and child cooperation in parent–child emotion dialogues, a second factor could be identified that was replicated across samples. This factor reflected parent and child strategies to constructively discuss more difficult feelings of children and come to a positive closure of these negative feelings by emphasizing a child’s strength and ability to competently deal with these feelings and situations. Some support was found for the predictive validity of these scales in child problem behavior and trauma symptoms. These results are a first step to identify dimensions in parent–child emotion dialogues using the AEED, that might refine our understanding of these dialogues. Further validation of the multidimensional structure may have clinical relevance in identifying targets for parent–child interventions. Future studies might take a closer look at the correlates of the factor closure/resolution as this factor may add a specific less explored facet of child-parent emotion dialogues.

## Supplemental Material

sj-docx-1-jiv-10.1177_08862605251322812 – Supplemental material for Dimensional Structure of Parent–Child Emotion Dialogues in Families Exposed to Interpersonal Violence: Associations with Internalizing, Externalizing and Trauma Symptoms Supplemental material, sj-docx-1-jiv-10.1177_08862605251322812 for Dimensional Structure of Parent–Child Emotion Dialogues in Families Exposed to Interpersonal Violence: Associations with Internalizing, Externalizing and Trauma Symptoms by J. C. de Schipper, M. M. Overbeek and M. H. M. de Moor in Journal of Interpersonal Violence
