# Supplementary material for: Dimensional Structure of Parent–Child Emotion Dialogues in Families Exposed to Interpersonal Violence: Associations with Internalizing, Externalizing and Trauma Symptoms
Source: J Interpers Violence. 2025 Mar 15;41(9-10):1763–85. doi: 10.1177/08862605251322812 (PMC13047221; doi:10.1177/08862605251322812)
Supplement: sj-docx-1-jiv-10.1177_08862605251322812 – Supplemental material for Dimensional Structure of Parent–Child Emotion Dialogues in Families Exposed to Interpersonal Violence: Associations with Internalizing, Externalizing and Trauma Symptoms [file sj-docx-1-jiv-10.1177_08862605251322812.docx]

**Dimensional Structure of Parent-Child Emotion Dialogues in Families Exposed to Interpersonal Violence: Associations with Internalizing, Externalizing and Trauma Symptoms**

**Supplementary Tables 1-6**

Supplementary Table 1

*Descriptives of the AEED Scales at Posttest in Sample 1 of Parent-Child Dyads Exposed to Interpersonal Trauma.*

|  | Sample 1 (*N* = 116) at Posttest | | |
| --- | --- | --- | --- |
|  | Min | Max | *M* (*SD*) |
| Parental Acceptance | 1.5 | 9 | 5.13 (1.41) |
| Parental Involvement | 2 | 8 | 4.98 (1.47) |
| Parental Structuring | 1 | 9 | 4.24 (1.61) |
| Parental Closure | 1 | 7.5 | 4.42 (1.34) |
| Parental Focus | 2 | 9 | 6.76 (1.91) |
| Parental Boundary Dissolution | 1 | 7.5 | 2.25 (1.64) |
| Parental Hostility | 1 | 7.5 | 1.26 (0.93) |
| Child Acceptance | 1 | 8.5 | 5.87 (1.45) |
| Child Cooperation | 1 | 9 | 5.36 (1.57) |
| Child Elaboration | 1 | 8 | 4.15 (2.02) |
| Child Resolution | 1 | 7 | 4.54 (1.02) |
| Child Focus | 3 | 9 | 7.80 (1.42) |
| Child Boundary Dissolution | 1 | 7 | 1.59 (1.21) |
| Child Hostility | 1 | 5 | 1.20 (0.59) |
| Adequacy | 1 | 9 | 4.42 (1.72) |
| Coherence | 1 | 8 | 3.94 (1.69) |

*Note.* Higher scores describe more enactment of the specific behavior.

Supplementary Table 2

*Descriptives of the AEED Scales in Subgroups of Sample 2: Parent-Child Dyads Exposed to Interpersonal Trauma (Clinical Group) and Dyads Not Exposed (Community Comparison Group).*

|  | Clinical group (*n* = 59) | | | Community Comparison group (*n*=59) | | |
| --- | --- | --- | --- | --- | --- | --- |
|  | Min | Max | *M* (*SD*) | Min | Max | *M* (*SD*) |
| Parental Acceptance ** | 2 | 8 | 5.27 (1.36) | 2 | 9 | 6.16 (1.71) |
| Parental Involvement *** | 2 | 8 | 4.81 (1.48) | 2 | 8 | 5.78 (1.48) |
| Parental Structuring *** | 2 | 7 | 3.81 (1.38) | 1 | 8 | 5.10 (1.66) |
| Parental Closure *** | 1 | 8 | 4.23 (1.25) | 1 | 8 | 5.20 (1.65) |
| Parental Focus | 4 | 9 | 7.54 (1.72) | 3 | 9 | 7.93 (1.22) |
| Parental Boundary Dissolution * | 1 | 7 | 2.22 (1.51) | 1 | 5 | 1.70 (0.99) |
| Parental Hostility | 1 | 6 | 1.22 (0.74) | 1 | 4 | 1.14 (0.51) |
| Child Acceptance *** | 2 | 8 | 5.48 (1.51) | 3 | 8 | 6.32 (1.46) |
| Child Cooperation *** | 2 | 8 | 4.79 (1.53) | 3 | 8 | 5.91 (1.55) |
| Child Elaboration *** | 1 | 7 | 2.97 (1.77) | 1 | 8 | 4.25 (1.99) |
| Child Resolution *** | 3 | 7 | 4.35 (0.93) | 2 | 8 | 5.17 (1.00) |
| Child Focus * | 2 | 9 | 7.76 (1.95) | 4 | 9 | 8.51 (1.06) |
| Child Boundary Dissolution | 1 | 7 | 2.02 (1.61) | 1 | 6 | 1.59 (1.22) |
| Child Hostility | 1 | 5 | 1.27 (0.74) | 1 | 3 | 1.15 (0.45) |
| Adequacy *** | 2 | 8 | 4.10 (1.66) | 2 | 9 | 5.29 (2.09) |
| Coherence *** | 1 | 7 | 3.20 (1.65) | 1 | 8 | 4.56 (1.94) |

*Note.* Higher scores describe more enactment of the specific behavior; *t*-test for mean differences between groups indicates: * *p*<.05; ** *p*<.01; *** *p*<.001.

Supplementary Table 3

*Pearson Correlations among AEED Subscales at Posttest in Sample 1 of Parent-Child Dyads Exposed to Interpersonal Trauma.*

|  | P Acc | P Inv | P Str | P Clo | P Foc | P BD | P Hos | C Acc | C Cop | C Elab | C Res | C Foc | C BD | C Hos | Adeq | Coh |
| --- | --- | --- | --- | --- | --- | --- | --- | --- | --- | --- | --- | --- | --- | --- | --- | --- |
| P Acc |  | .72 | .68 | .52 | .42 | .39 | .40 | .51 | .56 | .55 | .42 | .21 | .17 | .23 | .62 | .64 |
| P Inv |  |  | .83 | .59 | .40 | .39 | .19 | .45 | .58 | .52 | .50 | .16 | .27 | .11 | .52 | .64 |
| P Str |  |  |  | .56 | .23 | .31 | .12 | .48 | .59 | .68 | .50 | .12 | .16 | .10 | .67 | .72 |
| P Clo |  |  |  |  | .45 | .42 | .10 | .14 | .31 | .31 | .76 | .14 | .33 | .12 | .47 | .46 |
| P Foc |  |  |  |  |  | .60 | .32 | .17 | .20 | .17 | .31 | .41 | .51 | .32 | .23 | .23 |
| P BD |  |  |  |  |  |  | .20 | .17 | .24 | .16 | .27 | .22 | .39 | .29 | .21 | .24 |
| P Hos |  |  |  |  |  |  |  | .43 | .41 | .23 | .09 | .35 | .12 | .52 | .20 | .29 |
| C Acc |  |  |  |  |  |  |  |  | .83 | .61 | .19 | .23 | .08 | .36 | .56 | .66 |
| C Cop |  |  |  |  |  |  |  |  |  | .65 | .33 | .16 | .08 | .29 | .65 | .74 |
| C Elab |  |  |  |  |  |  |  |  |  |  | .31 | .17 | .08 | .19 | .82 | .83 |
| C Res |  |  |  |  |  |  |  |  |  |  |  | .15 | .15 | .05 | .44 | .45 |
| C Foc |  |  |  |  |  |  |  |  |  |  |  |  | .31 | .22 | .10 | .16 |
| C BD |  |  |  |  |  |  |  |  |  |  |  |  |  | .26 | .14 | .14 |
| C Hos |  |  |  |  |  |  |  |  |  |  |  |  |  |  | .22 | .16 |
| Adeq |  |  |  |  |  |  |  |  |  |  |  |  |  |  |  | .88 |
| Coh |  |  |  |  |  |  |  |  |  |  |  |  |  |  |  |  |

*Note.* P=Parental, C=Child, Acc=Acceptance, Inv=Involvement, Str=Structuring, Clo=Closure, Foc=Focus, BD=Boundary Solution, Hos=Hostility, Cop=Cooperation, Elab=Elaboration, Res=Resolution, Adeq=Adequacy, Coh=Coherence. Please note that BD and Hos subscale have been reverse coded. Dark grey: large to very large effect size (*r >* .50), medium grey: moderate-to-large effect size (.30 < *r* < .50), light grey: small-to-medium effect size (.10 < *r* < .30). All correlations of 0.18 or lower were not statistically significant (*p* > .05).

Supplementary Table 4

*Factor solution of the Principal Component Analysis of the AEED subscales at posttest in Sample 1 of parent-child dyads exposed to interpersonal trauma.*

|  | Sample 1 - Posttest | | |
| --- | --- | --- | --- |
|  | Factor 1 | Factor 2 | Factor 3 |
| Parental Acceptance | .65 | .28 | .12 |
| Parental Involvement | .63 | .37 | -.10 |
| Parental Structuring | .80 | .17 | -.18 |
| Parental Closure | .31 | .68 | -.24 |
| Parental Focus | -.12 | .82 | .38 |
| Parental Boundary Dissolution | -.04 | .73 | .24 |
| Parental Hostility | .19 | .09 | .74 |
| Child Acceptance | .78 | -.23 | .42 |
| Child Cooperation | .85 | -.14 | .27 |
| Child Elaboration | .91 | -.16 | .06 |
| Child Resolution | .38 | .49 | -.29 |
| Child Focus | -.08 | .40 | .52 |
| Child Boundary Dissolution | -.21 | .72 | .28 |
| Child Hostility | .05 | .20 | .72 |
| Adequacy | .89 | -.03 | -.01 |
| Coherence | .94 | -.04 | .02 |
| *Explained variance* | *42.93* | *13.66* | *11.25* |

Supplementary Table 5

*Factor solution of the Principal Component Analysis of the AEED subscales in the Clinical Subsample of Sample 2 of parent-child dyads exposed to interpersonal trauma.*

|  | Sample 2 - Clinical group (N = 59) | | | |  |
| --- | --- | --- | --- | --- | --- |
|  | Factor 1 | Factor 2 | Factor 3 | Factor 4 | Factor 5 |
| Parental Acceptance | .44 | .13 | -.04 | .62 | -.15 |
| Parental Involvement | .67 | .12 | .02 | .31 | .06 |
| Parental Structuring | .88 | .19 | .07 | .00 | -.09 |
| Parental Closure | .09 | .92 | .09 | .06 | .07 |
| Parental Focus | .02 | -.12 | .63 | .30 | .14 |
| Parental Boundary Dissolution | -.01 | .10 | .80 | .20 | -.17 |
| Parental Hostility | -.28 | -.02 | .10 | .93 | -.15 |
| Child Acceptance | .62 | -.13 | -.11 | .32 | .22 |
| Child Cooperation | .70 | -.09 | -.13 | .20 | .28 |
| Child Elaboration | .92 | -.12 | .06 | -.26 | .03 |
| Child Resolution | -.09 | .96 | -.07 | -.03 | .14 |
| Child Focus | -.03 | -.14 | .12 | -.08 | .83 |
| Child Boundary Dissolution | -.06 | .32 | -.05 | -.21 | .79 |
| Child Hostility | .06 | .00 | .80 | -.23 | .15 |
| Adequacy | 1.02 | .03 | .02 | -.23 | -.15 |
| Coherence | 1.04 | -.04 | .03 | -.23 | -.13 |
| *Explained variance* | *36.89* | *14.99* | *10.46* | *8.16* | *6.96* |

Supplementary Table 6

*Results of the Hierarchical Regression Analyses for the Replicated AEED Factors and the Maladaptive Scales Predicting Internalizing, Externalizing and Trauma Symptoms in the Two Samples Combined.*

| **Internalizing Symptoms** | B | | *SE* | | Beta | | *t* | | *p* | | *R2* | | *R2 change* | |
| --- | --- | --- | --- | --- | --- | --- | --- | --- | --- | --- | --- | --- | --- | --- |
| Step 3 |  | |  | |  | |  | |  | | .040 | | .020 | |
| Intercept | 2.58 | | 1.08 | |  | | 2.36 | | .019 | |  | |  | |
| Sensitive Guidance & Cooperation | .04 | | .08 | | .04 | | .47 | | .641 | |  | |  | |
| Closure/Resolution | -.17 | | .10 | | .13 | | -1.71 | | .090 | |  | |  | |
| Parental Focus | -.02 | | .08 | | -.02 | | -.27 | | .791 | |  | |  | |
| Parental Boundary Dissolution | .11 | | .08 | | .10 | | 1.28 | | .202 | |  | |  | |
| Parental Hostility | .14 | | .17 | | .06 | | .81 | | .417 | |  | |  | |
| Child Focus | .01 | | .10 | | .01 | | .15 | | .884 | |  | |  | |
| Child Boundary Dissolution | .00 | | .09 | | .00 | | .05 | | .962 | |  | |  | |
| Child Hostility | .08 | | .15 | | .04 | | .49 | | .672 | |  | |  | |
| **Externalizing Symptoms** | | B | | *SE* | | Beta | | *t* | | *p* | | *R2* | | *R2 Change* |
| Step 4 | |  | |  | |  | |  | |  | | .085* | | .030 |
| Intercept | | 4.69 | | .1.29 | |  | | 3.64 | | <.001 | |  | |  |
| Child Age | | -.14 | | .06 | | -.16 | | -2.30 | | .02 | |  | |  |
| Sensitive Guidance & Cooperation | | -.13 | | .09 | | -.12 | | -1.57 | | .12 | |  | |  |
| Closure/Resolution | | -.05 | | .11 | | -.03 | | -.43 | | .67 | |  | |  |
| Parental Focus | | .10 | | .08 | | .11 | | 1.32 | | .19 | |  | |  |
| Parental Hostility | | .33 | | .18 | | .14 | | 1.82 | | .07 | |  | |  |
| Child Focus | | -.17 | | .10 | | -.15 | | -1.67 | | .10 | |  | |  |
| Child Boundary Dissolution | | .01 | | .09 | | .00 | | .05 | | .96 | |  | |  |
| Child Hostility | | -.01 | | .16 | | -.00 | | -.04 | | .97 | |  | |  |
| Step 5 | |  | |  | |  | |  | |  | | .105** | | .020* |
| Intercept | | 4.04 | | .1.32 | |  | | 3.064 | | .00 | |  | |  |
| Child Age | | -.13 | | .06 | | -.15 | | -2.12 | | .04 | |  | |  |
| Sensitive Guidance & Cooperation | | -.11 | | .09 | | -.10 | | -1.31 | | .19 | |  | |  |
| Closure/Resolution | | -.04 | | .11 | | -.03 | | -.36 | | .72 | |  | |  |
| Parental Focus | | .15 | | .08 | | .16 | | 1.85 | | .07 | |  | |  |
| Parental Hostility | | .31 | | .18 | | .13 | | 1.70 | | .09 | |  | |  |
| Child Focus | | -.20 | | .10 | | -.17 | | -1.94 | | .05 | |  | |  |
| Child Boundary Dissolution | | -.06 | | .10 | | -.05 | | -.63 | | .53 | |  | |  |
| Child Hostility | | .04 | | .16 | | .02 | | .26 | | .80 | |  | |  |
| Parent Boundary Dissolution | | .19 | | .09 | | .16 | | 2.09 | | .04 | |  | |  |
| **Trauma Symptoms** | | B | | *SE* | | Beta | | *t* | | *p* | | *R2* | | *R2 change* |
| Step 3 | |  | |  | |  | |  | |  | | .056 | | .023 |
| Intercept | | .69 | | .20 | |  | | 3.40 | | <.001 | |  | |  |
| Sensitive Guidance & Cooperation | | -.01 | | .02 | | -.07 | | -.91 | | .36 | |  | |  |
| Closure/Resolution | | -.03 | | .02 | | -.13 | | -1.65 | | .10 | |  | |  |
| Parental Focus | | .01 | | .01 | | .04 | | .46 | | .65 | |  | |  |
| Parent Boundary Dissolution | | .03 | | .02 | | .14 | | 1.78 | | .08 | |  | |  |
| Parental Hostility | | .03 | | .03 | | .06 | | .84 | | .40 | |  | |  |
| Child Focus | | -.01 | | .02 | | -.07 | | -.75 | | .45 | |  | |  |
| Child Boundary Dissolution | | -.02 | | .02 | | -.07 | | -.89 | | .38 | |  | |  |
| Child Hostility | | -.01 | | .03 | | -.02 | | -.21 | | .84 | |  | |  |
